# Editorial: Oligodendrocytes: from their development to function and dysfunction

**DOI:** 10.3389/fncel.2024.1376931

**Published:** 2024-03-07

**Authors:** Shingo Miyata, Hiroaki Wake

**Affiliations:** ^1^Division of Molecular Brain Science, Research Institute of Traditional Asian Medicine, Kindai University, Osaka, Japan; ^2^Department of Anatomy and Molecular Cell Biology, Nagoya University Graduate School of Medicine, Nagoya, Japan

**Keywords:** oligodendrocytes, brain disease, neurodegeneration, oligodendrocyte progenitor cells, myelin, remyelination

Human central nervous system (CNS) myelination occurs before 20 years old and continues throughout our lives (Herbert and Monk, 2017). CNS myelination and remyelination by oligodendrocytes (OLs) is important for obtaining rapid conduction of action potentials and appropriate neuronal communications to support higher brain functions (Masson and Nait-Oumesmar, 2023). OLs and oligodendrocyte precursor cells (OPCs) exist in the corpus callosum, and OPCs have the ability to cell-divide and differentiate into OLs. Previous studies have examined various signal pathways of OL development, CNS myelination, and remyelination *in vivo* and *in vitro* analysis systems (Taylor and Monje, 2023). During CNS myelination and remyelination, OLs generate a multitude of processes and new myelin sheaths by wrapping suitable axons. However, the extent of involvement of various signal cascades and/or molecules in these developing OL lineage cells, CNS myelination, and remyelination remains to be fully elucidated. Thus, this Research Topic is looking to address key aspects of the function and dysfunction of OLs, promote the discussion around this Research Topic, and facilitate knowledge dissemination.

The co-editor, Wake's lab members Yoshida et al., report that the different properties of Ca^2+^ responses of OLs are induced activity-dependent glutamate and adenosine triphosphate (ATP) release from neurons or astrocytes. Further, these activity-dependent responses were lost in the Alzheimer's disease (AD) mice model, but a higher frequency of ATP release induced Ca^2+^ responses due to neurodegeneration. Hong et al. perform a systematic analysis of multiple brain regions and cerebrospinal fluid (CSF), and socially isolating dog groups during the juvenile stage led to a small number of differentially expressed genes in multiple brain regions except the prefrontal cortex (PFC). Maruyama et al. apply global lipidomic analyses to identify circulating lipids that mediate amyotrophic lateral sclerosis (ALS) pathogenesis. They identified a decrease in circulating free fatty acids, including oleic acid (OA) and linoleic acid (LA), and OA and LA inhibited excitotoxic oligodendrocyte cell death via the cell surface receptor FFAR1 (free fatty acid receptor1) in ALS model mice. Zhao et al. find that the OPC differentiation and OL morphology were significantly different between the brain and spinal cord, and inhibition of endoplasmic reticulum (ER) stress could effectively attenuate OPC death. Han et al. discuss recent findings suggesting an unexpected role of oligodendroglia, the cells that received far less attention than neurons and other glial cells. They also reviewed the possibility that OL lineage cells might be one of the most vulnerable cell types responding to the changing microenvironment in the brain during neurodegenerative diseases. Delfino et al. report that only platelet-derived growth factor receptor alpha (PDGFR-α) positive oligodendrocyte lineage cells are ciliated and reveal heterogeneity in the frequency of cilium presence on OPCs, depending on primary culture conditions and cerebral regions of mice. Further, they show the plasticity of oligodendroglia primary cilium length in response to different drugs. Mei et al. indicate the important molecular and genetic evidence that inositol 1,4,5-trisphosphate receptor type 2 (Itpr2) is dramatically up-regulated in differentiating OLs and regulates OL differentiation and myelin development through an extracellular signal-regulated kinase (ERK)-dependent mechanism. Valihrach et al. review the current understanding of OL heterogeneity in health and disease based on single-cell and single-nucleus transcriptomic technologies. They provide our OL research community with a unified overview of key transcriptomic studies dealing with OL heterogeneity in the mammalian CNS and consensus marker genes of selected OL populations. Chacon-De-La-Rocha et al. report that there is a premature decrease in OPC density at 9 months in AD model mice and that at 14 months, OPC displayed a shrunken and fibrous morphology, indicative of morphological dystrophy. They also indicate that changes in OPCs are potential factors in the progression of AD pathology. This Research Topic highlights the important themes of unraveling the mechanisms behind oligodendrocytes' formation and function, which may lead to a better understanding of their dysfunction and role in CNS pathologies.

## Author contributions

SM: Conceptualization, Writing—original draft. HW: Writing—review & editing.
